# Parental Knowledge Toward Meningococcal Disease and Vaccination in Makkah Region, Saudi Arabia: A Cross-Sectional Study

**DOI:** 10.7759/cureus.54450

**Published:** 2024-02-19

**Authors:** Ibrahim S Alibrahim, Abdulrahman I Khoj, Abdullah S Alibrahim, Khalid H Alnafei, Abrar A Alghamdi, Turki T Alessa, Abdulrahman M Alsuwayhiri, Naif M Almeqaty

**Affiliations:** 1 College of Medicine and Surgery, Umm Al-Qura University, Makkah, SAU; 2 Department of Pediatrics, King Fahd Hospital, Albaha, SAU; 3 Department of Pediatric Emergency, Maternity and Children Hospital, Makkah, SAU

**Keywords:** meningitis, invasive meningococcal disease, vaccination, parental, knowledge

## Abstract

Background

Invasive meningococcal disease (IMD) is a bacterial infection caused by Neisseria meningitidis, which primarily affects the meninges, with a high incidence in young children. The most effective technique for preventing IMD is vaccination, which has been available for over 40 years through meningococcal polysaccharide capsule-containing vaccines. This study aims to assess the parental knowledge of meningococcal disease and vaccination in the Makkah region of Saudi Arabia.

Methodology

A cross-sectional study was conducted between September and December 2023 among 597 parents in the Makkah region using a validated online survey. The collected data were analyzed using the Statistical Package for the Social Sciences (SPSS).

Results

The study sample included 597 parents, of which 339 (56.8%) were female and 258 (43.2%) were male. Our research demonstrated that 388 (65%) participants had an insufficient understanding of IMD, while 209 (35%) had a sufficient understanding. There was a significant correlation between the knowledge score and the completion of the routine vaccination and whether vaccinating a child is essential for the protection of other members of society.

Conclusions

Based on our study, only around one-third of the participants demonstrated a sufficient level of knowledge regarding IMD and its vaccination. To provide a more accurate assessment of the Saudi population, additional research should be conducted in various regions and cities.

## Introduction

Invasive meningococcal disease (IMD) is a bacterial infection caused by Neisseria meningitidis; it affects the meninges, which are the protective membranes that surround the brain and spinal cord [1]. Although IMD can affect people of all ages, young children have the highest incidence of the disease, followed by teenagers and young adults [2]. There are 13 different serogroups of meningococcal bacteria, which are classified based on the type of polysaccharide capsule they produce [3]. The capsule is the primary factor determining the bacteria’s virulence [3]. Six of these serogroups (A, B, C, W, X, and Y) are the most commonly associated with meningococcal disease [4]. Meningitis and septicemia are the two most prevalent manifestations of meningococcal infection [5]. The most effective technique for preventing IMD is vaccination, which has been available for over 40 years through meningococcal polysaccharide capsule-containing vaccines [6]. Throughout the 1990s, there was a significant and rapid increase in severe meningococcal C cases in European nations and Canada, which was accompanied by a shift toward hypervirulent C strains [7]. In response, several nations initiated vaccination programs with the new generation of the meningococcal C conjugate vaccine [7]. The estimated incidence of IMD worldwide is 1.2 million cases, with mortality rates ranging from 4% to 20% with effective management and reaching up to 80% in untreated patients [6]. In Saudi Arabia, between 1995 and 2011, the incidence of IMD was 1.103 cases, with the highest cumulative incidence among children younger than five years old [8]. Millions of people visit Makkah, Saudi Arabia, each year to do the Hajj and Umrah, creating one of the largest mass gatherings in the world [9]. Multiple Hajj-related IMD outbreaks among the local KSA population have been linked to large gatherings that follow the visitors of the Hajj and Umrah [8,10]. Despite the significant prevalence of meningococcal disease in Saudi Arabia, particularly among children, it is essential to assess the degree of knowledge and awareness among parents who are responsible for making decisions about the health and vaccines of their children [6]. A study conducted in Szczecin, Poland, revealed that 27.5% of the 350 participating parents had inadequate knowledge about IMD [3]. Another study conducted in the UK revealed that the majority of parents were not familiar with the term *meningococcal disease* [11]. Moreover, a study performed in Minnesota in the United States found that 24.5% of the 445 total parents were generally unaware of meningococcal disease vaccines [12]. To the best of our knowledge, no studies have been conducted among parents in Saudi Arabia. Therefore, this study aims to assess parents’ knowledge and awareness of meningococcal infections and vaccinations in the Makkah region of Saudi Arabia.

## Materials and methods

Participants

A total of 597 participants, with the majority (339, 56.8%) being females were recorded. The participants’ ages ranged from 18 to over 60. Among all the participants, 366 (61.3%) held at least a bachelor’s degree. In terms of their monthly income, 215 participants (36.6%) reported earning more than 10,000 Saudi Riyals (SR). The majority of the participants (308, 51.6%) depended upon healthcare workers as their source of medical information; 331 (55.4%) individuals were employed, while the remaining participants were unemployed and retired.

Procedure

This is a cross-sectional study investigating parental knowledge about IMD and vaccinations in the Makkah region of Saudi Arabia. OpenEpi version 3.0 was used to determine the minimum required sample size for this inquiry, factoring in the following aspects: the estimated population was around 8,325,304 individuals [13], with a 95% confidence interval (CI), and an expected frequency of 50%. The computed sample size was 384 participants, subsequently increasing to 400 to account for possible data loss. The study’s inclusion criteria included parents with children younger than 18 years old in the Makkah region. Participants who worked in the medical field and participants who did not agree to the study’s informed consent or who did not fulfill the participant criteria were excluded. Participants were recruited using a convenience sampling method. Data were collected from September to December 2023. We utilized a validated questionnaire from a previously published study [3]. All participants agreed to provide their informed consent before completing the study questionnaire, and all data were kept anonymous. Participants voluntarily completed an online self-administered questionnaire. We adopted the English version of the questionnaire, which was translated into Arabic by two independent bilingual translators who first translated the English version to Arabic and then performed a backward translation from Arabic to English. The questionnaire was designed using Google Forms and distributed via a link shared on social media platforms (Twitter, Facebook, WhatsApp, and Telegram). Completing the questionnaire typically took participants between three and four minutes. We distributed the questionnaire after obtaining ethical approval on September 4, 2023, from the Biomedical Research Ethics Committee of Umm Al-Qura University, Makkah, KSA (approval number HAPO-02 -K-012-2023-09-1709).

Measures

Sociodemographic Data

Participants were asked to provide information about their gender, age, education level, monthly income, source of medical information, and occupation. 

Knowledge of IMD Among Parents

The survey consisted of five multiple-choice questions that assessed parental knowledge about meningococcal disease. A correct answer was worth one point for each question. The aggregate of the right answers in this section determined the total knowledge score, which ranged from zero to five.

Parental Awareness Toward Vaccines

This section consisted of three multiple-choice questions to assess parental awareness about meningococcal vaccines.

Data analysis

Data were gathered, reviewed, and translated from the Arabic language into English. After the variables were coded, the data were entered into IBM SPSS Statistics for Windows, Version 26.0 (IBM Corp., Armonk, NY). Data were considered to be statistically significant at a *P*-value of ≤0.05. Regarding knowledge, any correct answer was given a 1-point score. Overall knowledge levels about IMD among parents were assessed by summing up discrete scores for different correct knowledge items. The overall knowledge score was categorized as an insufficient level of knowledge if the participant’s score was from 0 to 2 of the overall score, and a sufficient level of knowledge was considered to be applicable if the participant’s score was from 3 to 5 of the overall score. A descriptive analysis was performed using percentages and by prescribing frequency distributions for the study variables, including participants’ personal data. The chi-square test was used to assess the significant associations between sociodemographic factors and IMD knowledge.

## Results

A total of 901 participants agreed to participate in this study, and 304 were excluded from the study because they did not meet the inclusion criteria; 597 were included in this study. Regarding the participants' sociodemographic characteristics, 339 (56.8%) were females, and 258 (43.2%) were males. Sixty-three (10.6%) participants were aged between 18 and 25 years. While the majority of the participants (242, 47.2%) were aged between 26 and 40 years, 239 (40.0%) of the total participants were between 41 and 60 years, and only 13 (2.2%) were aged above 60 years. The majority of the participants (366, 61.3%) had a university-level education. Regarding the monthly income, a total of 215 (36.6%) participants had monthly incomes of more than 10,000 SR, while 119 (19.9%) had monthly incomes of less than 1,000 SR. Additionally, almost half of the participants (308, 51.6%) depended on healthcare workers as their source of medical information. Finally, 331 (55.4%) participants were employed (Table 1).

**Table 1:** Sociodemographic characteristics of study participants. SR, Saudi Riyal

Characteristics		Frequency	Percentage
Age (years)	18-25	63	10.6
	26-40	282	47.2
	41-60	239	40.0
	>60	13	2.2
Gender	Female	339	56.8
	Male	258	43.2
Educational level	Below university	179	30.0
	University	366	61.3
	Above university	52	8.7
Monthly income	<1,000 SR	119	19.9
	1,000-5,000 SR	124	20.8
	6,000-10,000 SR	139	23.3
	>10,000 SR	215	36.0
Source of medical information	Family and friends	57	9.5
	Internet articles	119	19.9
	Social media	113	18.9
	Healthcare workers	308	51.6
Occupation	Unemployed	211	35.3
	Employed	331	55.4
	Retired	55	9.2

Table 2 presents the participants’ answers to IMD knowledge-related questions. Only 103 (32%) were aware that IMD is caused by bacteria. In addition, 221 (37%) were aware that IMD is a life-threatening disease. Regarding the transmission of IMD, of the total participants, 147 (24.6%) were aware that droplets transmit bacterial meningitis. Moreover, 308 (51.6%) believed that bacterial meningitis could be prevented by vaccinations, and 223 (37.4%) believed that vaccinations against meningococci also protect against sepsis. However, regarding parental awareness regarding vaccines, 420 (70.4%) stated that the routine vaccination was complete. Finally, 518 (86.8%) believed that vaccinating a child is important for the protection of other society members.

**Table 2:** Participants’ knowledge of IMD and vaccines.

Items	Yes, *n* (%)	No, *n* (%)	I don’t know, *n* (%)
Invasive meningococcal disease (IMD) knowledge among parents			
IMD is caused by bacteria.	193 (32.3)	23 (3.9)	381 (63.8)
IMD is a life-threatening disease.	221 (37.0)	34 (5,7)	342 (57.3)
Bacterial meningitis is transmitted by droplets.	147 (24.6)	56 (9.4)	394 (66.0)
Bacterial meningitis could be prevented by vaccinations.	308 (51.6)	19 (3.2)	270 (45.2)
Vaccination against meningococci also protects against sepsis.	223 (37.4)	24 (4.0)	350 (58.6)
Parental awareness toward vaccines			
Is routine vaccination complete?	420 (70.4)	50 (8.4)	127 (21.3)
Vaccinating a child is important for other society members.	518 (86.8)	19 (3.2)	60 (10.1)
Do you have any concerns about vaccinating your children?	123 (20.6)	474 (79.4)	

**Figure 1:**
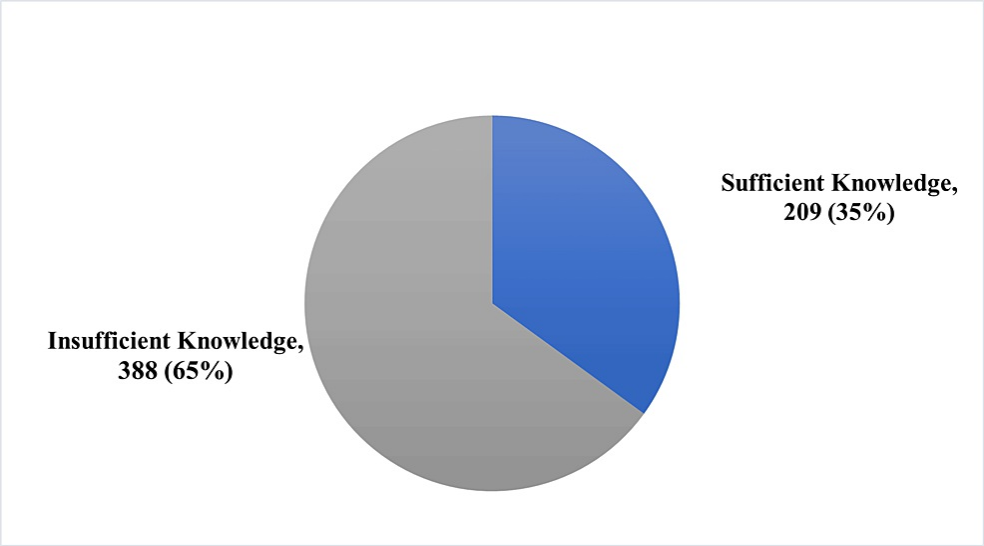
Level of knowledge of the participants toward meningococcal disease and vaccination.

The associations between IMD knowledge and sociodemographic factors were measured and are presented in Table 2. Participants were classified as having a sufficient level of knowledge and an insufficient level of knowledge based on the participant's correct answers. A total of 388 (65.0%) participants scored an insufficient level of knowledge, while 209 (35%) of participants scored a sufficient level of knowledge (Figure 1).

There was no significant association between knowledge score with age (0.389), gender (0.643), educational level (0.774), monthly income (0.057), source of medical information (0.591), and occupation (Table 3).

**Table 3:** Association of sociodemographic factors with IMD knowledge levels. IMD, invasive meningococcal disease

Sociodemographic factors	Total number of participants, *n* (%)	Knowledge level		*P*- value
		Insufficient knowledge	Sufficient knowledge	
Age (years)				
18-25	63 (10.6)	36 (57.1)	27 (42.9)	
26-40	282 (47.2)	184 (65.2)	98 (34.8)	0.389
41-60	239 (40)	161 (67.4)	78 (32.6)	
>60	13 (2.2)	7 (53.8)	6 (46.2)	
Gender				
Female	339 (56.8)	223 (65.8)	116 (34.2)	0.643
Male	258 (43.2)	165 (64.0)	93 (36.0)	
Educational level				0.774
Below university	179 (30.0)	120 (67.0)	59 (33.0)	
University	366 (61.3)	234 (63.9)	132 (36.1)	
Above university	52 (8.7)	34 (65.4)	18 (34.6)	
Monthly income (SR)				
<1,000	119 (19.9)	82 (68.9)	37 (31.1)	
1,000-5,000	124 (20.8)	70 (56.5)	54 (43.5)	0.057
6,000-10,000	139 (23.3)	86 (61.9)	52 (38.1)	
>10,000	215 (36.0)	150 (69.8)	65 (30.2)	
Source of medical information				
Family and friends	57 (9.5)	41 (71.9)	16 (28.1)	
Internet articles	119 (19.9)	76 (63.9)	43 (36.1)	0.591
Social media	113 (18.9)	76 (67.3)	37 (32.7)	
Healthcare workers	308 (51.6)	195 (63.3)	113 (36.7)	
Occupation				
Unemployed	211 (35.3)	147 (69.7)	64 (30.3)	0.204
Employed	331 (55.4)	206 (62.2)	125 (37.8)	
Retired	55 (9.2)	35 (63.6)	20 (36.4)	

There was a statistically significant association between the knowledge score and the completion of routine vaccinations (<0.001) and their belief that vaccinating a child is vital for the protection of other society members (<0.001) (Table 4).

**Table 4:** Association of parental awareness toward vaccines with IMD knowledge levels. IMD, invasive meningococcal disease

Variable	Total number of participants, *n* (%)		Knowledge level, *n* (%)		*P*-value
			Insufficient knowledge	Sufficient knowledge	
Is routine vaccination complete?					
No	50 (8.4)		27 (54.0)	23 (46.0)	<0.001
Yes	420 (70.4)		259 (61.7)	161 (38.3)	
I don’t know	127 (21.3)		102 (80.3)	25 (19.7)	
Vaccinating a child is important for other society members					
No		19 (3.2)	17 (89.5)	2 (10.5)	<0.001
Yes		518 (86.8)	319 (61.6)	199 (38.4)	
I don’t know		60 (10.1)	52 (86.7)	8 (13.3)	
Do you have any concerns about vaccinating your children?					
No		474 (79.4)	311 (65.6)	163 (34.4)	0.533
Yes		123 (20.6)	77 (62.6)	46 (37.4)	

## Discussion

This study aimed to determine the level of knowledge about IMD and its vaccination among parents in the Makkah region in Saudi Arabia, in addition to assessing the relationship between IMD knowledge and the socio-demographic data. Raising public awareness about IMD and its vaccination is crucial in preventing the disease [14,15]. Unfortunately, our study demonstrates that only 209 (35%) parents possessed sufficient knowledge regarding IMD, while the remaining had insufficient knowledge. The current study indicates a decline in knowledge levels; conversely, in a similar study conducted in Poland, two-thirds of the participants had a sufficient level of knowledge about IMD [3]. Moreover, in Australia, a community-based study found that 63.4% of participants possessed a strong degree of knowledge about meningococcal disease [16]. One possible reason for this decrease in knowledge in our study might be that 179 (30%) participants did not finish their university educations. A second explanation for this knowledge decline may be the fact that nearly half of the participants rely on social media, family, friends, and online self-search rather than healthcare workers as sources of medical information. According to our survey, 289 (48.4%) participants did not think or know that vaccinations can prevent bacterial meningitis. In contrast, the study in Poland revealed that only 20.3% did not believe that vaccinations can prevent bacterial meningitis [3]. Furthermore, a study conducted in Lithuania demonstrated that one-third of the sample refused to give their children vaccinations, with the majority of them believing it is unsafe [17]. This illustrates the need for meningococcal vaccination health education campaigns among parents. In this study, 177 (29.7%) participants did not complete the routine vaccination or did not know about it. In comparison, a study conducted in Sudair, Saudi Arabia, revealed that only 3.3% of the participants did not follow the Ministry of Health’s recommended vaccination schedule [18]. Likewise, another study conducted among Turkish parents found that only 3.4% of the children had not received their recommended vaccinations [19]. Although the advantages of child immunization are widely recognized, parents may decide to skip or delay the vaccines due to various potential concerns [20]. This reality emphasizes the necessity of more effective child immunization-related education during routine clinic visits to alleviate these concerns. Surprisingly, we found no significant correlation between the knowledge level and parents’ age, educational level, monthly income, source of medical information, and occupation. On the other hand, the Polish study found a strong correlation between knowledge score, marital status, and educational achievement [3]. Nevertheless, our research revealed a correlation between the degree of knowledge and the completion of routine vaccination and beliefs about whether vaccinating a child is crucial for the protection of community members. Childhood vaccination is essential to achieve community protection against infectious diseases. Community protection is particularly substantial to protect those who are unable to receive vaccines, such as immunocompetent patients and those below the scheduled age for the vaccine [21].

Study limitations

Although this study is the first in the Middle East to investigate parents’ levels of knowledge regarding IMD, it has some limitations. There were two possible limitations in the current study. First, there is a possibility of misunderstandings due to the use of a self-administered survey. Second, to compare the results of the participants properly, their age, gender, and educational achievement levels should be as similar as possible.

## Conclusions

In conclusion, our study aimed to assess IMD-related knowledge levels among parents. We found that only 209 (35%) participants had adequate levels of knowledge about meningococcal disease and vaccination, which indicates a lack of knowledge in the entire region. Our results suggest a significant association between parents who completed their children’s routine vaccination and parents who believe that vaccinating a child is vital for society. Our study did not find any significant association between the level of knowledge and sociodemographic factors. Additional research should be conducted in various locations, encompassing different cities and regions, to more accurately assess the status of Saudis.
